# Vascular risk factors and astrocytic marker for the glymphatic system activity

**DOI:** 10.1007/s11547-023-01675-w

**Published:** 2023-07-18

**Authors:** Yi-Chia Wei, Chih-Chin Heather Hsu, Wen-Yi Huang, Chemin Lin, Chih-Ken Chen, Yao-Liang Chen, Pin-Yuan Chen, Yu-Chiau Shyu, Ching-Po Lin

**Affiliations:** 1 Department of Neurology, Chang Gung Memorial Hospital, Keelung, Taiwan; 2 Community Medicine Research Center, Chang Gung Memorial Hospital, Keelung, Taiwan; 3 Institute of Neuroscience, National Yang Ming Chiao Tung University, Taipei, Taiwan; 4 College of Medicine, Chang Gung University, Taoyuan, Taiwan; 5 Center of Geriatrics and Gerontology, Taipei Veterans General Hospital, Taipei, Taiwan; 6 Department of Psychiatry, Chang Gung Memorial Hospital, Keelung, Taiwan; 7 Department of Radiology, Chang Gung Memorial Hospital, Keelung, Taiwan; 8 Department of Medical Imaging and Radiological Sciences, Chang Gung University, Taoyuan, Taiwan; 9 Department of Nursing, Chang Gung University of Science and Technology, Taoyuan, Taiwan; 10 Department of Education and Research, Taipei City Hospital, Taipei, Taiwan; 11 Department of Neurosurgery, Chang Gung Memorial Hospital, Keelung, Taiwan

**Keywords:** Glymphatic system, Astrocyte, GFAP, Diffusion tensor imaging, DTI-ALPS

## Abstract

**Objectives:**

Glymphatic system maintains brain fluid circulation via active transportation of astrocytic aquaporin-4 in perivascular space. The diffusion tensor imaging analysis along the perivascular space (DTI-ALPS) is an established method measuring perivascular glymphatic activity, but comprehensive investigations into its influential factors are lacking.

**Methods:**

Community-dwelling older adults underwent brain MRI scans, neuropsychiatric, and multi-domain assessments. Blood biomarker tests included glial fibrillary acidic protein (GFAP) for astrocyte injury.

**Results:**

In 71 enrolled participants, the DTI-ALPS index was associated with modifiable factors, including lipid profile (high-density lipoprotein, *r* = 0.396; very-low-density lipoprotein, *r* =  − 0.342), glucose intolerance (diabetes mellitus, standardized mean difference (SMD) = 0.7662; glycated hemoglobin, *r* =  − 0.324), obesity (body mass index, *r* =  − 0.295; waist, *r* =  − 0.455), metabolic syndrome (SMD =   − 0.6068), cigarette-smoking (SMD =  − 0.6292), and renal clearance (creatinine, *r* =  − 0.387; blood urea nitrogen, *r* =  − 0.303). Unmodifiable associative factors of DTI-ALPS were age (*r* =  − 0.434) and sex (SMD = 1.0769) (all *p* < 0.05).

A correlation of DTI-ALPS and blood GFAP was noticed (*r* =  − 0.201, one-tailed t-test for the assumption that astrocytic injury impaired glymphatic activity, *p* = 0.046). Their cognitive correlations diverged, domain-specific for DTI-ALPS (Facial Memory Test, *r* = 0.272, *p* = 0.022) but global cognition-related for blood GFAP (MoCA, *r* =  − 0.264, *p* = 0.026; ADAS-cog, *r* = 0.304, *p* = 0.010).

**Conclusion:**

This correlation analysis revealed multiple modifiable and unmodifiable association factors to the glymphatic image marker. The DTI-ALPS index correlated with various metabolic factors that are known to increase the risk of vascular diseases such as atherosclerosis. Furthermore, the DTI-ALPS index was associated with renal indices, and this connection might be a link of water regulation between the two systems. In addition, the astrocytic biomarker, plasma GFAP, might be a potential marker of the glymphatic system; however, more research is needed to confirm its effectiveness.

**Supplementary Information:**

The online version contains supplementary material available at 10.1007/s11547-023-01675-w.

## Introduction

### Glymphatic system

The glymphatic system maintains fluid circulation in the brain parenchyma and keeps cerebral waste washout, nurturing, and homeostasis. Because water permeability is forbidden by the blood–brain barrier (BBB), fluid cannot exchange freely between blood circulation and the brain fluid system. Therefore, brain fluid transportation is established from arterial perivascular space through the interstitial space to venous perivascular space. This system requires an active control system, which relies on astrocytes and the active water channel aquaporin 4 (AQP4) on their end-foot and, therefore, is named the *glymphatic system* [1].

### Conditions with glymphatic dysfunction

The glymphatic system keeps waste clearance from the brain, and glymphatic dysfunction is associated with cognitive decline and the accumulation of neurodegenerative proteins [2]. Besides pathological neurodegeneration, an increased prevalence of hypertension, small vessel disease, neurodegenerative protein deposition, and sleep dysfunction also predispose to glymphatic dysfunction in normal aging [3–5]. These pathological conditions are related to multiple mechanisms, including changes in BBB permeability, astrocytic injury, losing AQP4 polarization, loss of vascular plasticity, and neuroinflammation. The term “central nervous system interstitial fluidopathy” has been proposed to describe diseases with abnormal interstitial fluid dynamics. These diseases include Alzheimer’s disease, Parkinson’s disease, idiopathic normal pressure hydrocephalus, and cerebral small vessel disease [6, 7].

Nevertheless, even in physiological conditions, several factors influence glymphatic function (e.g., sleep cycle, cardiac pump, respiration, and neuronal activities). Besides, unmodifiable factors, like age and sex, also affect the levels of glymphatic activity [8].

### Image markers of the glymphatic system

To study the glymphatic system in humans, noninvasive imaging approaches are crucial to measuring glymphatic system activity in vivo [9], and previous observation of diffusivity changes along the perivascular space direction and its cognitive correlation suggested the feasibility of using diffusion imaging to measure the activity of the glymphatic system [10].

The diffusion tensor imaging analysis along the perivascular space (DTI-ALPS) index is a non-invasive imaging marker of the glymphatic system with good reproducibility [11], coupling with sleep-cycle dynamics [12], and sensitivity to reflect disease-related glymphatic dysfunction [10, 13]. The utilities of DTI-ALPS for measuring glymphatic system activity was also validated by a concurrent study of standard methods of intrathecal radioactive traces injection and diffusion MRI scans; its results showed good correlations between the classical contrast-enhanced MRI method with intrathecal administration of gadolinium agent and the DTI-ALPS by non-contrast diffusion MRI in calculating glymphatic clearance rate (*r *=   − 0.772 ~   − 0.844, *p* < 0.001) [14].

### Other potential markers of the glymphatic system

The astrocyte is the crucial operational unit of the glymphatic system. It controls the active transport of interstitial fluid via active water transportation to regulate transcellular fluid flow and affect glymphatic function [1]. Glial fibrillary acidic protein (GFAP) is an intermediate filament structural protein with high specificity for astroglia linage [15]. It is drained from the brain’s interstitial space to the blood by the glymphatic system [16]. Therefore, we propose testing whether blood GFAP can be a novel glymphatic marker.

### Study aims

This study aims to explore the factors influencing glymphatic function because these influential factors might be the key to improving glymphatic function. To this end, we used diffusion MRI to extract the glymphatic image marker, DTI-ALPS. We also conducted multidomain assessments and blood biomarker tests to investigate the effects of demographics, astrocytic and neuronal injury, vascular factors, and sleep habits on DTI-ALPS.

We also test if blood GFAP can be a glymphatic marker. A proper glymphatic marker should reflect cognition-maintaining function of the glymphatic system. Therefore, we examine the correlations between the glymphatic markers and cognitive performance.

## Methods

### Study design and participants

This study was joint with the Northeastern Taiwan Community Medicine Research Cohort (NTCMRC; ClinicalTrials.gov identifier NCT04839796) under the management of the Community Medicine Research Center of the Keelung Chang Gung Memorial Hospital. We enrolled non-demented healthy older adults aged between 60 and 80 from January 2020 to January 2022. Participants must meet the following criteria: (1) having no dementia, (2) independent of daily living, (3) having no major organ failure, (4) having no history of stroke, epilepsy, neuroinflammatory, neuroinfectious, neurodegenerative, or neurodevelopmental diseases, (5) no brain trauma or surgery history, and (6) no active psychiatric disease. Quality control of image preprocessing further excluded those patients with inadequate image quality or found to have brain lesions in the MRI images.

### MRI analysis

#### Image acquisition

MRI scans were performed on a Siemens 3 T Skyra MR scanner at Chang Gung Memorial Hospital, Keelung, Taiwan. All participants received identical pulse sequences, including a high-resolution 3D T1-weighted image (T1w) and a set of diffusion-weighted images (DWIs). The details of the acquisitions are available in eAppendix 1.

#### Image preprocessing

MRI data were processed using the iDIO pipeline [17] (https://github.com/iDIO4dMRI/iDIO) that included functions of MRtrix3 (https://www.mrtrix.org/), FSL (https://fsl.fmrib.ox.ac.uk/), ANTs (http://stnava.github.io/ANTs/), and PreQual (https://github.com/MASILab/PreQual). In brief, the DWIs underwent image denoising, Gibbs ringing removal, susceptibility-induced distortion, eddy current and subject movement correction, B1 field inhomogeneity correction, and imaging resampling to 2 mm^3^ isotropic voxels. T1w image preprocessing includes Gibbs-ringing artifact removal and B1 field inhomogeneity bias correction, followed by skull stripping. Preprocessed DWIs with b-values 1500 s/mm^2^ are extracted and analyzed using the diffusion tensor model with weighted least squares. The details of the image preprocessing steps are available in eAppendix1.

#### Diffusion tensor image analysis along the perivascular space (DTI-ALPS)

The DTI-ALPS, which estimates the diffusivity along the perivascular space of medullary veins at the level of the lateral ventricle in the left hemisphere, was used to evaluate the glymphatic activity of each participant [10]. The DTI-ALPS index measures the ratio of the mean of the x-axis diffusivity in the area of projection fibers (Dxxproj) and x-axis diffusivity in the area of the association fibers (Dxxassoc) to the mean of the y-axis diffusivity in the area of projection fibers (Dyyproj) and the z-axis diffusivity of the association fibers (Dzzassoc).

$${\text{DTI-ALPS}}\;{\text{index}} = \frac{{{\text{mean }}\left( {{\text{Dxxproj}},{\text{ Dxxassoc}}} \right)}}{{{\text{mean }}\left( {{\text{Dyyproj}},{\text{ Dzzassoc}}} \right)}}$$

An atlas-based approach was used to calculate the DTI-ALPS index [13]. The fractional anisotropy (FA) map of each participant was deformably co-registered to the FA map template of the ICBM-DTI-81 atlas [18]. The tensor map was warped to standard space based on the registration of the FA map. All the above steps are achieved by ANTs (ANTs command: antsRegistrationSyNQuick and antsApplyTransforms) and the accuracy of the registration was confirmed visually. The projection fibers (superior and posterior corona radiata) and association fibers (superior longitudinal fasciculus) were identified based on the ICBM-DTI-81 atlas labels with a limited x-axis range that embedded the passing direction of the vessels in the deep white matter. A mask with FA > 0.2 was also applied to avoid including CSF-occupied voxels. The DTI-ALPS index was then computed automatically.

### Immunomagnetic reduction for blood-based biomarkers for astrocytic injury, axonal damage, and neurodegeneration

Immunomagnetic reduction (IMR) detected trace amounts of molecules at a 10 pg/mL level in human plasma by utilizing the magnetic susceptibility changes upon the antibody-antigen complex formation on antibody-precoating magnetic nanoparticles. A superconducting quantum interference device (SQUID) magnetometer was used to measure the alternating-current magnetic susceptibility to quantify the target protein concentration in the sample [19]. Its low limit of detection is comparable with the standard method, single-molecule array (SIMOA), for neurodegenerative proteins in human blood [20].

The sample preparation protocol for blood-based biomarkers was identical for all participants as follows. The peripheral venous blood was collected in vacutainers coated with ethylenediaminetetraacetic acid (EDTA). The blood tubes were gently inverted for mixture and then centrifuged at room temperature for 1500–2500 g for 15 min. The supernatant as the plasma was separated into Eppendorf tubes and stored at  − 80 ℃ before IMR analysis.

GFAP IMR reagents (MF-GFA-0060) were prepared using anti-GFAP antibodies (837204, Biolegend) covalently conjugated on dextran-coated magnetic Fe_3_O_4_ nanoparticles (MF-DEX-0060, MagQu). The magnetic beads and antibodies were stored in phosphate-buffered saline; the final concentration was 10 mg Fe/ml. A 60 μl of GFAP IMR reagent was mixed with a 60 μl plasma, and the analysis was performed on an IMR analyzer (XacPro-S, MagQu).

Neurofilament light chain (NfL) is a structural protein of axons in neurons and is used as a neuronal axonal injury marker. The analyzing process of plasma NfL level was identical for a mixture of 60 μl NfL reagent (MF-NFL-0060, MagQu) and 60 μl plasma sample [21]. Plasma level of beta-amyloid 42 (Aβ42) and 40 (Aβ40), total tau (Tau), and phosphorylated tau 181 (p-Tau181) were also acquired from the IMR assays [22]. The concentrations of each sample were the mean values of the duplicate measurements.

### Blood tests for metabolic, vascular, and inflammatory factors

The blood tests for potential influential factors included renal function, electrolytes, liver enzyme, hemogram, blood sugar, insulin resistance, lipid profile, uric acid, folate, vitamin B12, vitamin D level, and acute inflammatory protein (e.g., high-sensitivity C-reactive protein).

### Demographics and lifestyle profile

The study recorded the demographic features, including age, sex, school education level, occupation, marriage, living environment, and financial status of the participants. The lifestyle and hobbies profiling queried cigarette smoking, alcohol drinking, tea (including flavored, bottled, and brewed tea) and coffee use.

The diagnoses of diabetes mellitus (DM) and hypertension were based on their past medical history and medication in use. Chronic kidney disease (CKD) was defined when 1) estimated glomerular filtration rate (eGFR) < 60 or 2) the presence of proteinuria by urine protein ≥ 1 + , albumin-creatinine ratio ≥ 30, or protein-creatinine ratio ≥ 150. Being overweight was defined by a body mass index (BMI) ≥ 25. Metabolic syndrome was defined by at least three items positive in five criteria, which were 1) waist circumference ≥ 90 cm in males and ≥ 80 cm in females, 2) systolic blood pressure ≥ 130 mmHg or diastolic blood pressure ≥ 85 mmHg, 3) fasting sugar (sugar AC) ≥ 100 mg/dL, 4) high-density lipoprotein (HDL) < 40 mg/dL in males and < 50 mg/dL in females, and 5) blood triglyceride (TG) ≥ 150 mg/dL [23].

### Cognitive tests

Participants are individually interviewed during the neuropsychiatric interview and the cognitive tests in a quiet, non-disturbed room by a well-trained researcher. Two structured cognitive tests used for global cognition assessments were the traditional Chinese version of the Montreal Cognitive Assessment (MoCA) [24] and the cognitive subscale of the Alzheimer’s Disease Assessment Scale (ADAS-cog) [25]. The other individual tests of specific cognitive domains were the Category Fluency (CF) [26], the Facial Memory Test (FMT), and three tests from the Wechsler Adult Intelligence Scale (WAIS-III), which were the Digit Symbol Substitution Test (DSST), the Digit Span Test forward (DST-f) and backward (DST-b), and the Letter Number Sequencing (LNS) [27].

### Mental assessment

The Mini-International Neuropsychiatric Interview (MINI) was a formal neuropsychiatric interview, used to exclude participants with active psychiatric disorders [28]. The depression and anxiety levels were assessed by the Hospital Anxiety and Depression Scale (HADS) [29].

### Physical activity assessment

Daily physical activeness was assessed by the International Physical Activity Questionnaire-Short From (IPAQ-SF) [30]. The results of IPAQ-SF were displayed in the estimated equivalent quantity of energy consumption out of daily physical activity in metabolic equivalent (MET minutes per week, IPAQ-SF MET).

### Sleep profiling

Sleep quality was queried by the Pittsburg Sleep Quality Index (PSQI) and the Epworth Sleepiness Scale (ESS) [31]. In addition, the participants who agreed to record sleep stages wore the SOMNOwatch™ (SOMNOmedics, Randersacker, Germany) for a 72 h recording [32].

### Statistical analysis

We first check the normality of the DTI-ALPS index in the enrolled participants. Then, comparisons of the DTI-ALPS index of binary groups used an independent t-test. Standardized mean difference (SMD) showed the group difference of the DTI-ALPS index in a standardized value. In addition, Pearson correlation analysis examined the correlations between the multidomain factors and the DTI-ALPS index. A one-tailed t-test was applied only on the specific occasion that assumed astrocytic injury impaired glymphatic activity, and plasma GFAP and the DTI-ALPS had a negative correlation. Besides, statistical significance was defined by a two-tailed *p*-value < 0.05. In addition, a false discovery rate (FDR) correlation was used to examine the results of multiple comparisons between the DTI-ALPS and multidomain factors. Statistical analysis was performed on R for Mac (https://www.r-project.org) and SPSS version 23 (IBM Corporation, USA).

## Results

### Participants, demographics, laboratory results, and multidomain assessments

**Fig. 1:**
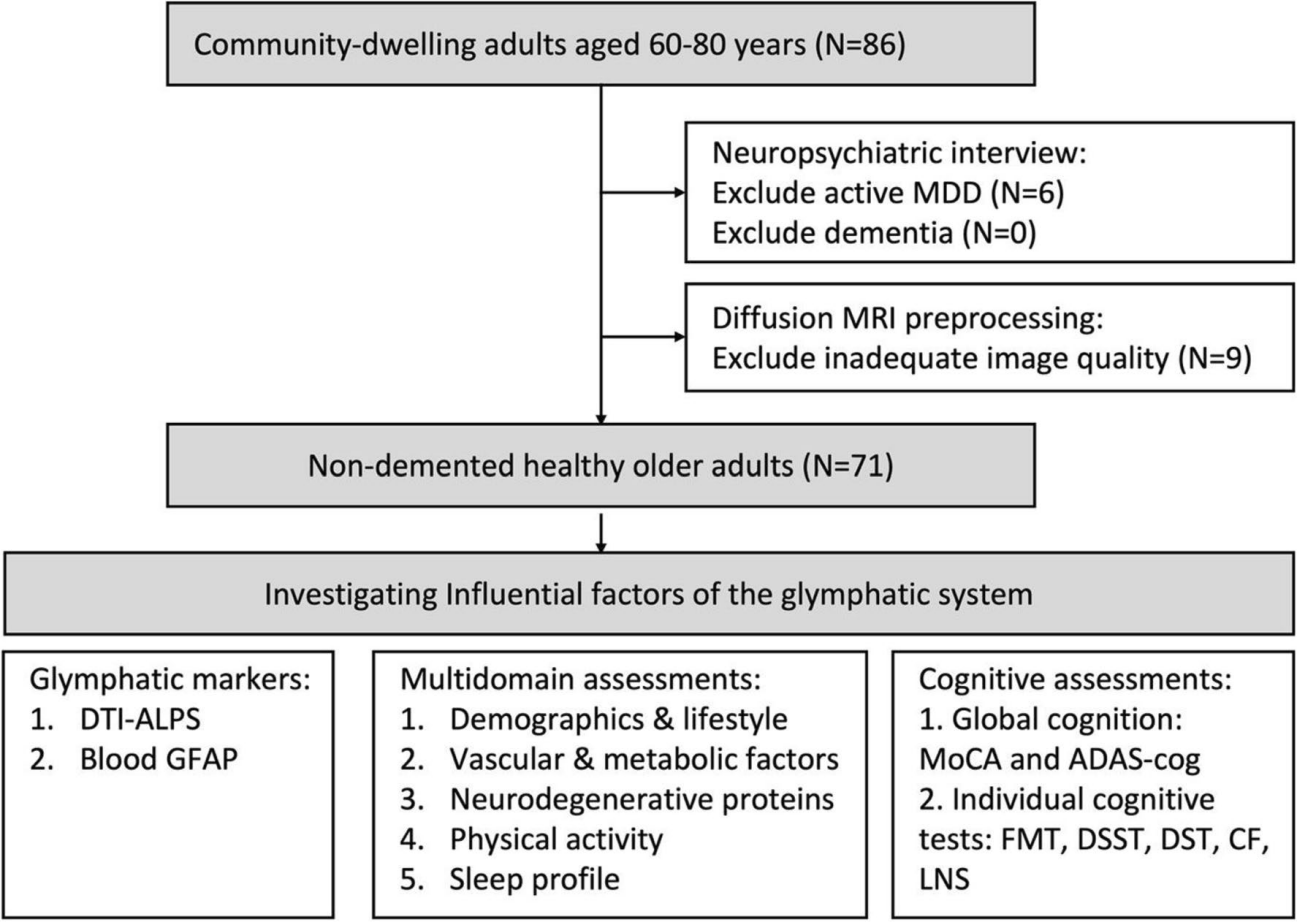
Flowchart of enrollment. Participants were enrolled from the communities, joining the Northeastern Taiwan community medicine research cohort. After neuropsychiatric interviews and brain MRI studies, the participants with active psychiatric disease, dementia, and inadequate brain image quality were excluded. From 86 participants for initial screening, 71 were enrolled for further investigation to identify influential factors of glymphatic function. MDD, major depressive disorders; DTI-ALPS, diffusion tensor image analysis along the perivascular space; GFAP, glial fibrillary acidic protein; MoCA, Montreal cognitive assessment; ADAS-cog, Alzheimer’s disease assessment scale; FMT, facial memory test; DSST, digit symbol substitution test; DST, digit span test; CF, category fluency; LNS, letter number sequencing

A total of 86 participants aged between 60 and 80 years were screened for eligibility. Six participants with active major depressive disorders were excluded from the neuropsychiatric interview. The MRI quality checkup further excluded one participant for image setting error, one for ill-placed field-of-view, and seven for motion artifacts with > 3% motion in total DWI volumes. A final of 71 participants underwent further studies (Fig. 1).

The mean age of enrolled participants was 67.65 ± 3.75 years old, and 62% (44 of 71) of them were female The complete demographics, glymphatic markers, laboratory data, results of multidomain assessments, and cognitive tests were summarized in eAppendix2.

### Correlations to the glymphatic image marker DTI-ALPS

The mean DTI-ALPS index was 1.24 ± 0.10. Normality checkup using the Kolmogorov–Smirnov test showed a normal distribution of the DTI-ALPS index of the 71 enrolled participants (D = 0.076, *p* = 0.200, which did not reject the null hypothesis that the variable follows a normal distribution). Therefore, we applied parametric statistic methods for further analysis.

**Fig. 2:**
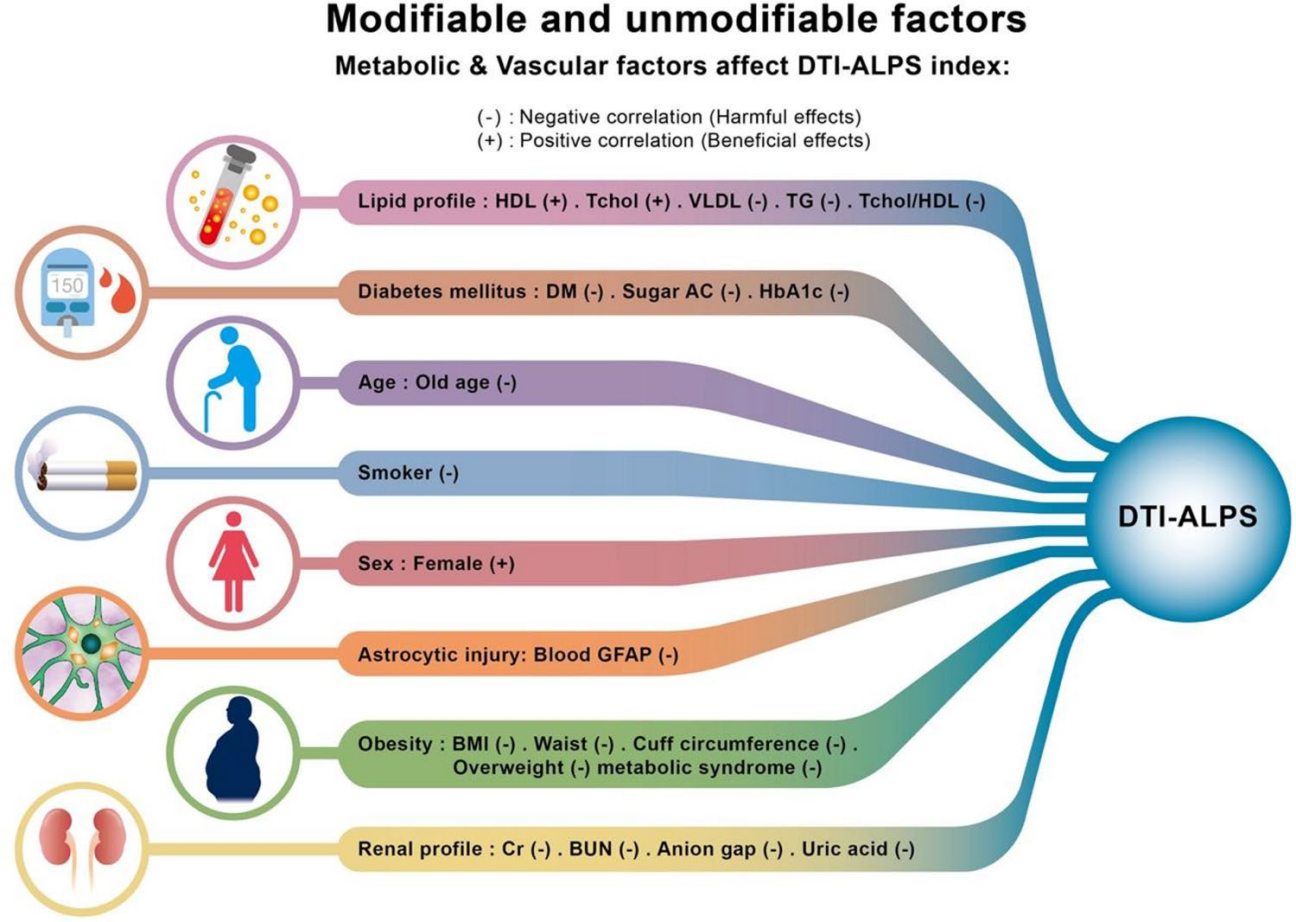
Factors associated with the glymphatic image marker DTI-ALPS index. Multidomain assessments revealed vascular and metabolic factors in association with DTI-ALPS. Renal clearance and anion balance was also associated. Some other factors were unmodifiable, including age and sex. Besides, the astrocytic injury marker, blood GFAP, showed weak correlations with DTI-ALPS. This correlation indicated the connections of the cellular and image markers of the astrocyte-centered glymphatic system. DTI-ALPS, diffusion tensor imaging analysis along the perivascular space; HDL, high-density lipoprotein; Tchol, total cholesterol; VLDL, very-low-density lipoprotein; TG, triglyceride; Tchol/HDL, ratio of Tchol and HDL; DM, diabetes mellitus; sugar AC, Ante Cibum blood sugar (fasting blood sugar); HbA1c, glycated hemoglobin; GFAP, glial fibrillary acidic protein; BMI, body mass index; Cr, creatinine; BUN, blood urea nitrogen

The Pearson correlation coefficients of the continuous parameters with the DTI-ALPS index are displayed in eTable1 (eAppendix2). The group comparisons of the DTI-ALPS index for the nominal variables are shown in eTable2 (eAppendix2). The above association analysis of the DTI-ALPS index and multidomain assessments revealed potentially influential factors of glymphatic activity (Fig. 2). Furthermore, after conducting the analysis for multiple comparisons, the following variables passed the FDR correction: age, sex, body weight, waist, creatinine, uric acid, triglyceride, HDL, and VLDL.

### Vascular and metabolic factors

The beneficial factors with positive correlations with the DTI-ALPS index for a facilitated glymphatic system activity were HDL (*r* = 0.292,* p* = 0.015), total cholesterol (Tchol) (*r* = 0.396,* p* = 0.001), blood phosphate level (P) (*r* = 0.300,* p* = 0.012), and female sex (SMD = 1.0769, *p* < 0.001).

The adverse factors that might hamper the glymphatic activity and with negative correlations to the DTI-ALPS index were triglyceride (TG) (*r* = − 0.342,* p* = 0.004), very-low-density (VLDL) (*r* = − 0.342,* p* = 0.004), the cardiac risk index by the ratio of total cholesterol and HDL (Tchol/HDL) (*r* = − 0.277,* p* = 0.021), fasting blood sugar (glucose AC) (*r* = − 0.246,* p* = 0.028), glycated hemoglobin (HbA1c) (*r* = − 0.324,* p* = 0.007), body weight (*r* = − 0.348,* p* = 0.003), BMI (*r* = − 0.295,* p* = 0.014), waist (*r* = − 0.455,* p* < 0.001) and cuff circumference (*r* = − 0.280,* p* = 0.028). The conditions associated with a lower DTI-ALPS index were overweight with a BMI over 25 (SMD = − 0.5818, *p* = 0.018), DM (SMD = − 0.7662, *p* = 0.018), metabolic syndrome (SMD = − 0.6068, *p* = 0.033), and cigarette smoking (SMD = − 0.6292, *p* = 0.020).

### Renal clearance

Besides, several renal function parameters showed negative effects with DTI-ALPS, which were creatinine (Cr) (*r* = − 0.387,* p* = 0.001), blood urea nitrogen (BUN) (*r* = − 0.303,* p* = 0.011), estimated anion gap (*r* = − 0.322,* p* = 0.007); however, neither the eGFR (*p* = 0.364) nor the BUN-to-Cr ratio (BUN/Cr ratio) (*p* = 0.685) was associated with DTI-ALPS. Besides, blood uric acid also showed a significant negative correlation with DTI-ALPS (*r* = − 0.381,* p* = 0.001).

### Astrocytic injury marker

**Fig. 3:**
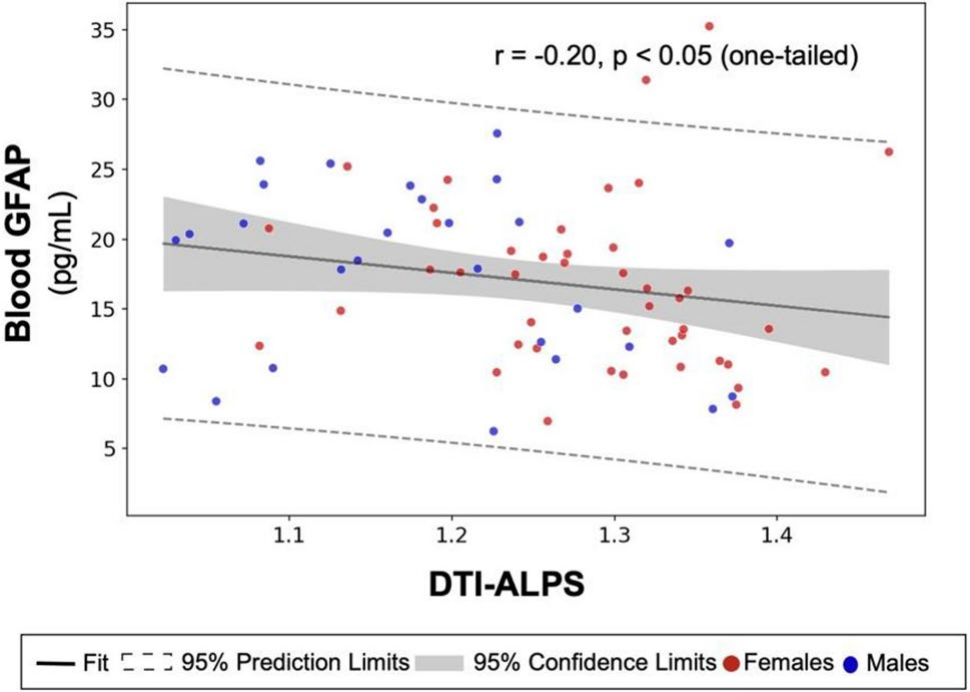
Correlation between the glymphatic image marker and blood-based astrocytic injury marker (DTI-ALPS vs. blood GFAP). Astrocyte is the main operating cell using the active water transporter AQP4 on their end-foot for facilitating brain fluid flow in the glymphatic system. GFAP is an astrocyte-specific cellular marker whose release into blood marks astrocytic injury. Under the assumption that astrocytic injury hampered glymphatic transportation, we found that elevated blood GFAP was weakly associated with decreased DTI-ALPS (Pearson’s correlation coefficient *r* =  − 0.201, one-tailed *p* = 0.046)

Under the assumption that astrocyte is the key operator of the active brain interstitial fluid transportation in and out of the perivascular space via AQP4 on astrocytic end-foot, increased blood GFAP for reactive astrogliosis might be accompanied by glymphatic dysfunction and a lower DTI-ALPS index [33]. The results showed a negative association between DTI-ALPS index and blood GFAP (*r* = − 0.201, one-tailed *p* = 0.046; Fig. 3). No significant associations with blood levels of other neurodegenerative proteins (NfL, Aβ42, Aβ40, Tau, and p-Tau181) (eAppendix2).

### Age effects

**Fig. 4:**
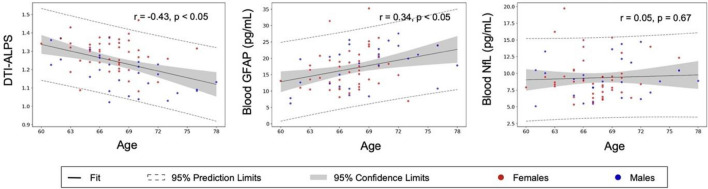
Significant age effects on DTI-ALPS and blood GFAP, but not on blood NfL. Blood neurofilament light chain (NfL) estimated the degree of axonal injury of the nervous system, blood GFAP represented astrocytic injury, and DTI-ALPS was the known glymphatic marker from brain diffusion MRI. Age oppositely impacted DTI-ALPS and blood GFAP but did not affect blood NfL

Old age was an unmodifiable risk factor for decreased DTI-ALPS (*r* = − 0.434,* p* < 0.001). In clarifying the age effects on astrocytes and neurons, we found that age significantly correlated with the astrocytic injury markers (blood GFAP, *r* = 0.34, *p* < 0.05) but was not relevant to the neuronal axonal injury marker (blood NfL, *r* = 0.05, *p* = 0.34) (Fig. 4).

### Mental, physical activity, and sleep factors

The questionnaire-based mental assessments found that a higher anxiety degree was associated with increased glymphatic system activity (HADS-A vs. DTI-ALPS, *r* = 0.238, *p* = 0.046). In contrast, physical activity was not related to DTI-ALPS (IPAQ-SF MET vs. DTI-ALPS, *p* = 0.390). The sleep assessment found a high degree of excessive daytime sleepiness was associated with higher DTI-ALPS (ESS vs. DTI-ALPS, *r* = 0.278, *p* = 0.019); however, sleep quality was not associated with DTI-ALPS (PSQI vs. DTI-ALPS, *p* = 0.104) (eAppdendix2).

Otherwise, a total of 35 participants completed the 72-h SOMNOwatch recording. As the non-rapid eye movement (NREM) sleep period was already known to be the time of active glymphatic transportation [8, 34], however, the ratio of non-REM and rapid eye movement (REM) sleep failed to show a significant correlation with the DTI-ALPS index (NREM/REM ratio vs. DTI-ALPS, *p* = 0.078) (eAppdendix2).

### Cognitive correlations of the glymphatic markers

**Fig. 5:**
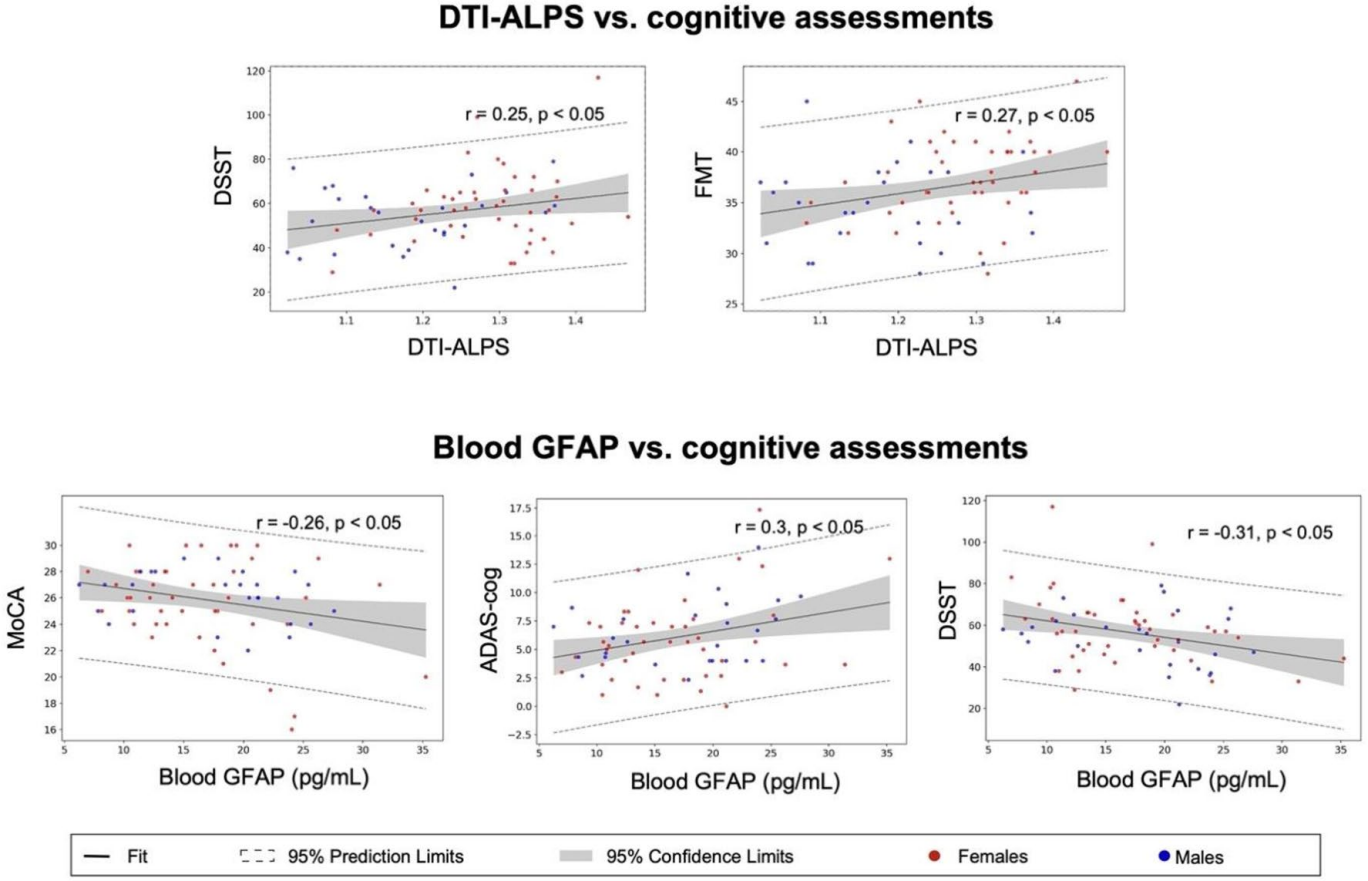
Different cognitive assessments correlated with DTI-ALPS and blood GFAP. A higher glymphatic activity by a greater DTI-ALPS index was associated with better cognitive performance in DSST and FMT. A lower blood GFAP level was noticed in the participants who performed well in MoCA, ADAS-cog (scores represent deductions), and DSST. Statistical significance at a two-tailed* p* < 0.05. DSST, Digit Symbol Substitution Test; FMT, Facial Memory Test; MoCA, Montreal Cognitive Assessment; ADAS-cog, the cognitive subscale of the Alzheimer’s Disease Assessment Scale

DTI-ALPS and blood GFAP were significantly associated with different cognitive test scores. A higher DTI-ALPS index correlated with better performance in DSST (*r* = 0.248, *p* = 0.037) and FMT (*r* = 0.272, *p* = 0.022). There was no significant correlation between DTI-ALPS and other cognitive assessments. On the other hand, a lower blood GFAP level was significantly associated with better performance in MoCA (*r* = − 0.264, *p* = 0.026), ADAS-cog (*r* = 0.304, *p* = 0.010), and DSST (*r* = − 0.309, *p* = 0.009) (Fig. 5).

In addition, a further investigation of blood GFAP levels found positive associations with older age (*r* = 0.336, two-tailed *p* = 0.004), longer REM sleep by minutes (*r* = 0.406, *p* = 0.016), and a higher percentage of REM sleep in total sleep time (*r* = 0.398, *p* = 0.018). In contrast, a higher school education level (*r* = − 0.255, *p* = 0.032) and a higher blood vitamin B12 level (*r* = − 0.268, *p* = 0.026) were protective from astrocytic injury with negative correlations with blood GFAP (eAppdendix2).

**Fig. 6:**
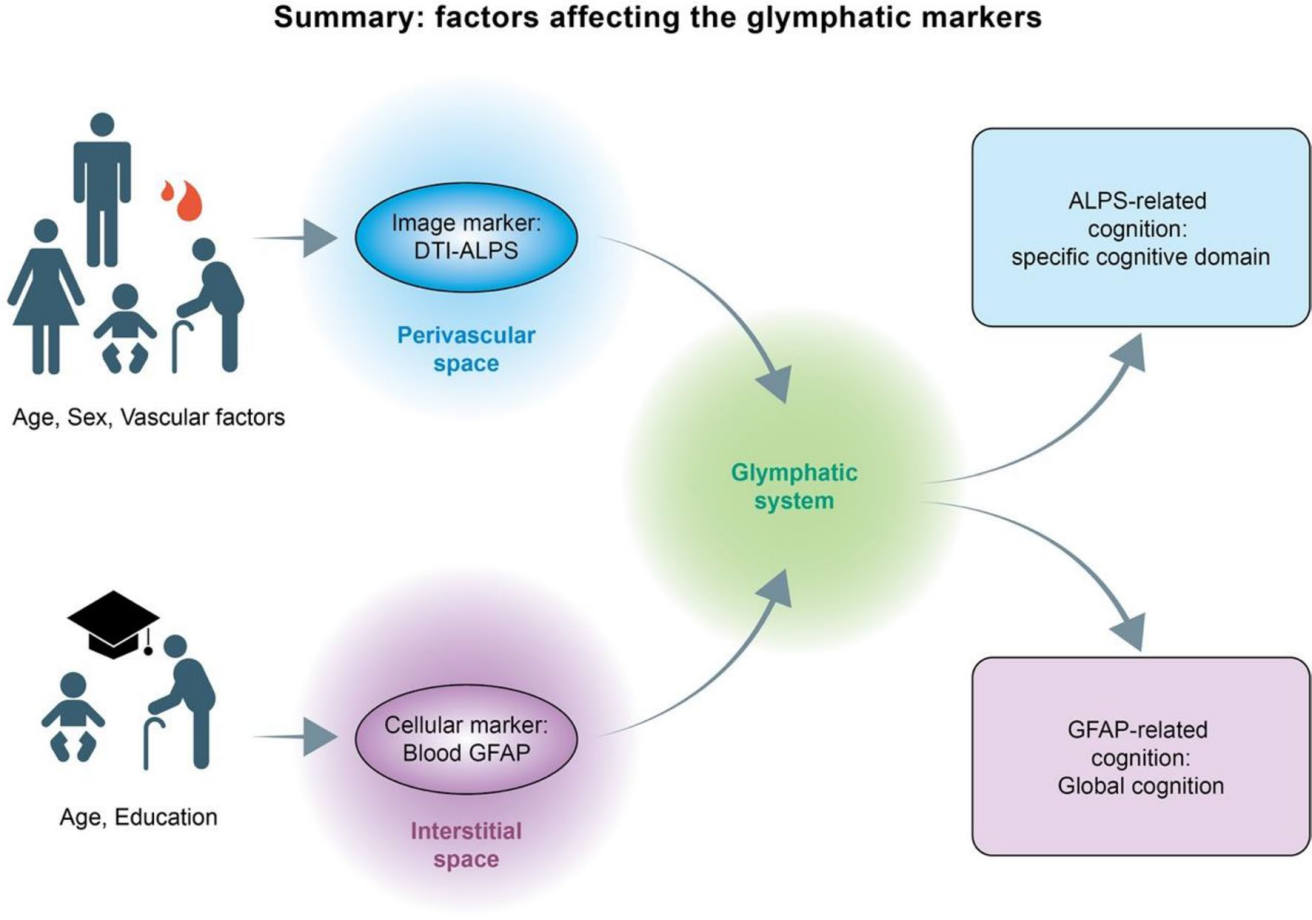
Cognitive correlations with the glymphatic markers. The DTI-ALPS measures the glymphatic activity of perivascular space in diffusion MRI images, and blood GFAP tests the release of astrocytic structural protein from the interstitial space of brain parenchyma. These markers correlated with different cognitive domains: global cognition with blood GFAP and specific domains with DTI-ALPS. In addition, these two glymphatic markers had different associative factors: sex, vascular, and metabolic factors affect DTI-ALPS, but education was associated with blood GFAP. Besides, age had common effects on both glymphatic factors

Therefore, we summarized the divergent cognitive domain associations, different influential factors, and physiological significance of the DTI-ALPS index and blood GFAP in Fig. 6.

## Discussion

### Summary

This study explored influential factors of glymphatic function by performing correlation analysis between the glymphatic image marker DTI-ALPS and multidomain assessments in community-dwelling cognitively normal older adults. We found unmodifiable factors associated with DTI-ALPS, like age and sex. However, we also identified multiple vascular, metabolic, and renal factors associated with the DTI-ALPS index. The combo of influential factors highlights the importance of vascular conditions and water metabolism in balancing glymphatic dynamics.

In addition, the blood-based biomarker of astrocyte injury, blood GFAP, showed weak correlations with the DTI-ALPS index. Both DTI-ALPS and blood GFAP showed clinical correlations with cognitive performance but in divergent domains. Blood GFAP also had different associative factors other than those of DTI-ALPS. This similarity in essence but the difference in characteristics indicates the value of blood GFAP as a novel glymphatic marker representing different physiological meanings from those of the DTI-ALPS index.

### Modifiable and unmodifiable factors of glymphatic function

Animal and human studies have established the physiological model of glymphatic regulation. For example, sleep stages, respiration, cardiac pulsation, and intracranial oscillations affect glymphatic dynamics [8]. In contrast, altered vascular elasticity or BBB permeability impairs glymphatic function. In this case, glymphatic activity is decreased in spontaneously hypertensive rats and diabetic rats [4, 35].

In investigating glymphatic system's influential factors, we found a close relationship between good glymphatic function and vascular healthiness; a higher DTI-ALPS index is related to a lower risk of atherosclerosis and endothelial injury. First, the dual effects of lipid profiles on the DTI-ALPS index echo the counterpart mechanism of VLDL and HDL on endothelial dysfunction [36]. VLDL is a liver-secreted triglyceride and cholesterol-containing larger particle that predisposes endothelial injury and atherosclerosis, elevates insulin resistance, and might impaired glymphatic activity. In contrast, HDL maintains vessel wall clearance to possibly keep neurovascular component in the glymphatic system unobstructed [37]. Next, glucose intolerance induces endothelial dysfunction via increased reactive oxidative and nitrogen spices. The endothelial permeability and endothelial relaxation are impaired and might reduce glymphatic function and impede interstitial fluid flow, resulting in a lower DTI-ALPS index [38].

Notably, the diagnosis of metabolic syndrome summed up blood pressure, lipid profile, glucose intolerance, and obesity and was associated with a lower DTI-ALPS in a recent study comparing older adults with and without metabolic syndrome [39]. Similarly, our study showed the group difference in community-dwelling healthy older adults (eTable 2) and further identified correlations of the individual components of metabolic syndrome with the DTI-ALPS index, including TG, HDL, fasting sugar, and waist circumference (Fig. 2 and eTable1). In summary, the glymphatic system is not isolated in the brain but is affected by systemic metabolism, possibly related to the general wellness of the microvascular environment.

The molecular mechanism of renal clearance is similar to that of glymphatic clearance that relies on the active water transporter, aquaporins. With evidence from bench to clinical, impairment of aquaporin function leads to kidney diseases [40]. Impaired glymphatic activity was observed in chronic kidney disease, from mild to advanced stages [41, 42]. Besides, our results also support this renal-brain association in water metabolism that blood Cr and BUN correlated with the DTI-ALPS index. However, more studies of aquaporin polymorphism and post-translational modification of these water channel families are required to confirm this renal-glymphatic linkage.

Beside the above modifiable factors, age is an unmodifiable factor that increases glymphatic dysfunction. We found an increase in blood GFAP and a decrease in DTI-ALPS with older age. Other studies also support these age effects on the glymphatic system. Zhou et al. observed a decline in glymphatic clearance rate after intrathecal contrast agent injection with age advanced in older adults [43]. In the other postmortem human study, aging was accompanied by alternations in AQP4 expression and loss of AQP4 polarization, which were related to Aβ deposition [44]. Lastly, increased prevalence of hypertension, small vessel disease, Aβ deposition, and sleep dysfunction with age have been proposed to predispose aging-related glymphatic dysfunction [3–5].

### Proposing blood GFAP to be a novel glymphatic marker

The astrocytic structural protein GFAP is released into brain interstitial space, flowing into CSF, and drained by the glymphatic system into peripheral blood in astrogliosis [16]. The CSF GFAP level correlates with its blood level, and blood GFAP represents astrocyte reactivity and astrocyte injury in the central nervous system [45, 46]. For example, the aging process alters astrocytic morphology and molecular expression. Under oxidative stress and chronic low-grade inflammation, astrocytes become reactive, and GFAP is upregulated and released from aging astrocytes [47].

The correlation between DTI-ALPS and blood GFAP represents astrocyte-centered image-cellular coupling. Several in vivo studies used diffusion MRI metrics to simulate the effects of AQP4 on brain tissue water diffusion. For example, altered diffusion MRI signals reflect astrocyte changes in animal models post-AQP4 manipulation. After RNA interference knocked down AQP4 expression acutely, impaired astrocytic water transportation was observed, along with an subsequent reduction in the apparent diffusion coefficient [48]. AQP4 inhibitor injection also significantly lowered water diffusivity in brain parenchyma and changed diffusion indices in MRI images [49].

**Fig. 7:**
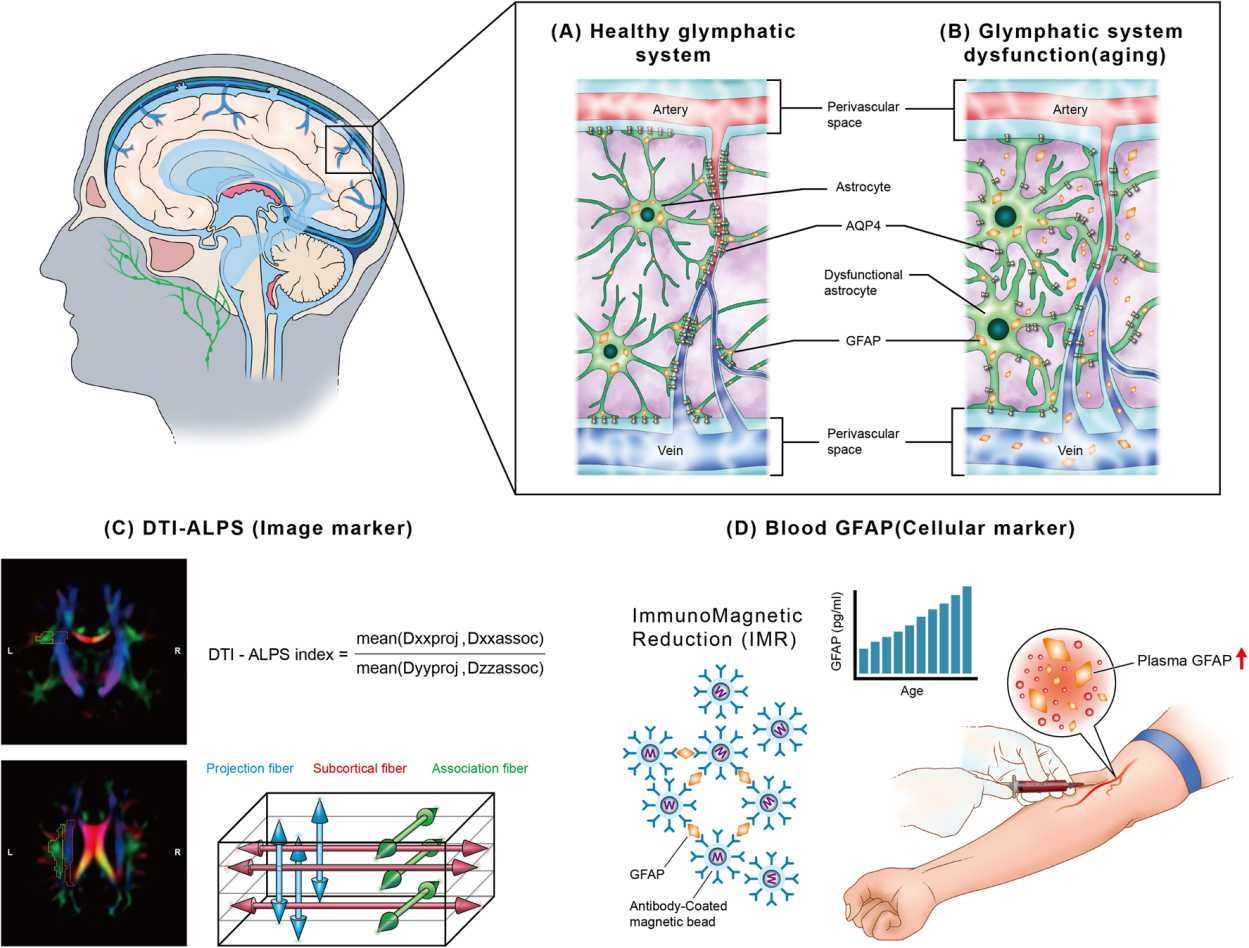
Summary of the markers for glymphatic system activity. **A** The glymphatic system is the center of brain fluid transportation, controlled by active water transportation through the AQP4 water channel on astrocytic end-feet. Operated by the glymphatic system, cerebrospinal fluid influxes to the interstitial space, mixes with the interstitial fluid, and then effluxes to the cerebral venous and lymphatic drainage system. **B** In aging process, function of the glymphatic system decreases with less effective wastes clearance. In addition, astrocyte morphological changes, astrogliosis, and increased release of GFAP into the interstitial fluid occur with aging. Then the GFAP in brain fluid is drained into blood, and the blood GFAP level increases correspondently. **C** DTI-ALPS is a non-invasive method to track the glymphatic dynamics in the brain by measuring water diffusivity of perivascular space on diffusion MRI. **D** We proposed that blood GFAP could be a cellular marker of the glymphatic system and used the immunomagnetic reduction technique to measure the concentration of GFAP in plasma. In summary, DTI-ALPS (the imaging marker) correlated with blood GFAP (the cellular marker) in community-dwelling older adults and correlated with their cognitive performance. Blood GFAP can help estimate the healthiness of the glymphatic system in the brain. DTI-ALPS, diffusion tensor imaging analysis along the perivascular space; GFAP, glial fibrillary acidic protein; AQP4, aquaporin 4; Dxxproj, the x-axis diffusivity in the area of projection fibers; Dxxassoc, the x-axis diffusivity in the area of the association fibers; Dyyproj, the y-axis diffusivity in the area of projection fibers; Dzzassoc, the z-axis diffusivity of the association fibers

In summary, we proposed blood GFAP to be a cellular marker of the glymphatic system based on the AQP4-astrocyte model for brain interstitial fluid active transportation, the correlations with DTI-ALPS index, association with cognitive performance, and non-invasive accessibility (Fig. 7).

### Divergent cognitive correlations to DTI-ALPS and blood GFAP

The DTI-ALPS index was more particularly associated with specific cognitive functions (DSST and FMT), but blood GFAP was related more to global cognitive performance (MoCA, ADAS-cog, and DSST). The divergent correlations might have underlain physiological significance. First, GFAP release from brain astrocytes to the blood depends on the glymphatic system in a multi-step manipulation experiment using a mouse model to study the transport route of the biomarker from the brain to the blood [16]. Therefore, blood GFAP reflects the collective astrocytic injury of the whole brain without regional specificity, which may explain why blood GFAP correlates with global cognition.

In contrast, the cognitive correlates of the DTI-ALPS index were domain-specific in this study. Even though the method of measuring DTI-ALPS tries to eliminate the effects of nerve fiber bundles by using sites that are perpendicular to them, however, it has still been affected by their influence, possibly due to the angle of intersection between major fiber bundles and the perivascular space not always being exactly 90 degrees. When evaluating the impact of glymphatic system activity on cognitive function using the DTI-ALPS, it's important to consider how regional brain specialization can affect the representative value of the index. The calculation of the DTI-ALPS index was based on diffusion metrics of selected periventricular white matter regions of interest (ROIs) along the medullary veins (Fig. 7C) [13]. The vessels run laterally to the paracentral gyrus, supramarginal gyrus, middle cingulum, and caudate [10]. These brain regions are involved in specific cognitive functions such as cognitive flexibility [50], feedback-mediated cognitive control [51], and language-receiving process. Moreover, these regions are in the middle of facial perception and memory networks [52]. Maybe because of the topological closeness to cognitive-functioning areas, the DTI-ALPS index correlated with the specific cognitive test scores of DSST and FMT.

### Limitations

There are several limitations to the current study that need to be addressed. First, as the image and cellular marker were relatively non-invasive indirect methods to capture the glymphatic dynamics and estimate glymphatic activity, the evidence levels are limited and less robust than those experiments of AQP4 knockout mice and intrathecal contrast enhancement images.

Second, the current DTI-ALPS method considered the directional dependency between projection fiber, association fiber, and medullary veins. Therefore, the periventricular area of the lateral ventricle became the only place where we can partially evaluate the diffusivity along the perivascular space. To determine whether the DTI-ALPS index is region-dependent to specific cognitive function, methods for assessing diffusivity along the perivascular area in other brain regions may be needed in future studies.

Third, although the association between blood GFAP and DTI-ALPS index was noticed, we did not use a disease model to test this cellular marker. Future studies are expected to validate the glymphatic markers in diseases with impaired glymphatic function, like Alzheimer’s disease, multiple sclerosis, and stroke.

## Conclusion

The image marker DTI-ALPS for the glymphatic system is linked to both modifiable factors, including lipid and glucose metabolism, obesity, metabolic syndrome, and renal clearance, and unmodifiable factors, such as sex and aging. These risk factors are commonly associated with vascular diseases like atherosclerosis. The glymphatic activity seems vulnerable to these factors as it passes through the perivascular spaces, which can be influenced by the microenvironment. Additionally, it appears that there may be an association between the renal indices and the DTI-ALPS index, indicating a possible linked mechanism of water metabolism in both the renal and glymphatic systems. Besides, although the current evidence is insufficient, plasma GFAP might be a potential biomarker of the glymphatic system based on the principal role of aquaporin-mediated active transportation of astrocytes in this system. Finally, by conducting this correlation analysis, we hope to bring out more investigation to gain insight into the pathophysiology of the glymphatic system and glimpse the potential future of more understanding of diseases with impaired glymphatic function.

## Supplementary Information

Below is the link to the electronic supplementary material.

Supplementary file1 (PDF 93 KB)

Supplementary file2 (PDF 140 KB)

## Data Availability

Anonymized data not published within this article will be made available by request from qualified investigators.

## Abbreviation

Aβ: Beta-amyloid
ADAS-cog: The cognitive subscale of the Alzheimer’s disease assessment scale
AQP4: Aquaporin 4
BBB: Blood–brain barrier
BMI: Body mass index
BUN: Blood urea nitrogen
CF: Category fluency
CKD: Chronic kidney disease
Cr: Creatinine
DM: Diabetes mellitus
DSST: Digit symbol substitution test
DST-f: Digit span test forward
DST-b: Digit span test backward
DTI-ALPS: Diffusion tensor imaging analysis along the perivascular space
DWI: Diffusion-weighted image
Dxxassoc: X-axis diffusivity in the area of the association fibers
Dxxproj: X-axis diffusivity in the area of projection fibers
Dyyproj: Y-axis diffusivity in the area of projection fibers
Dzzassoc: Z-axis diffusivity of the association fibers
EDTA: Ethylenediaminetetraacetic acid
eGFR: Estimated glomerular filtration rate
ESS: Epworth sleepiness scale
FA: Fractional anisotropy
FMT: Facial memory test
GFAP: Glial fibrillary acidic protein
HbA1c: Glycated hemoglobin
HADS: Hospital anxiety and depression scale
HDL: High-density lipoprotein
IMR: Immunomagnetic reduction
IPAQ-SF: International physical activity questionnaire-short from
IPAQ-SF MET: IPAQ-SF daily physical activity in metabolic equivalent
LNS: Letter number sequencing
MDD: Major depressive disorders
MINI: Mini-international neuropsychiatric interview
MoCA: Montreal cognitive assessment
NfL: Neurofilament light chain
NREM: Non-rapid eye movement
NTCMRC: Northeastern Taiwan community medicine research cohort
PSQI: Pittsburg sleep quality index
p-Tau181: Phosphorylated Tau 181
REM: Rapid eye movement
ROI: Region of interest
SIMOA: Single-molecule array
SQUID: Superconducting quantum interference device
Sugar AC: Ante cibum blood sugar (fasting blood sugar)
T1w: T1-Weighted image
Tau: Total Tau
Tchol: Total cholesterol
Tchol/HDL: Ratio of total cholesterol to high-density lipoprotein
TG: Triglyceride
VLDL: Very-low-density lipoprotein
WAIS-III: Wechsler adult intelligence scale III
