# Supplementary material for: Vascular risk factors and astrocytic marker for the glymphatic system activity
Source: Radiol Med. 2023 Jul 18;128(9):1148–61. doi: 10.1007/s11547-023-01675-w (PMC10474179; doi:10.1007/s11547-023-01675-w)
Supplement: Supplementary file 1 — Supplementary file1 (PDF 93 KB) [file 11547_2023_1675_MOESM1_ESM.pdf]

eAppendix 1

Cognitive test

MoCA was developed in 2005 for detecting early cognitive impairment[1] and was well-validated in Taiwan.[2] It contained structured tasks covering orientation, executive function, visuospatial skills, attention, calculation, working memory, delayed recall, and language for a global evaluation of cognition. The total score ranged from 0 (worst) to 30 (best) with an additive one-point correction for education years ≤ 12 years.[1] A cutoff score of 23/24 was suggested by the Taiwan version of MoCA for identifying mild cognitive impairment (MCI) with 92% sensitivity and 78% specificity.[2]

ADAS-cog was invented in 1984 for cognitive assessment of mild to severe Alzheimer's dementia.[3] It evaluated global cognition by 11 items for memory, praxis, and memory domains, with a total score from 0 (best) to 70 (worst). The Taiwan version of ADAS-cog in traditional Chinese had been validated in community-dwelling older adults.[4]

Image acquisition

MRI scans were performed on a Siemens 3T Skyra MR scanner equipped with a 20-channel head and neck coil at Chang Gung Memorial Hospital, Keelung, Taiwan. All participants received identical pulse sequences, including a set of high-resolution T1-weighted image (T1w) using a 3D Magnetization-Prepared Rapid Gradient Echo (MPRAGE) sequence (TR/TE = 2200/2.45 ms, TI = 900 ms, field-of-view (FOV) = 256 mm ×256 mm^2, flip angle = 8°, matrix size = 256 × 256, 176 sagittal slices, slice thickness = 1 mm, no gap). The MR scans also collected a set of diffusion-weighted images (DWIs) using single-shot spin-echo echo-planar diffusion-weighted images (TR/TE = 8500/99 ms, matrix size = 108 × 108, 50 contiguous slices, voxel size = 2.37 × 2.37 × 2.4 mm^3) with two b-values of 1500 s/mm^2 (30 diffusion directions) and 3000 s/mm2 (64 diffusion directions), plus two b=0 images in posterior to anterior phase encoding direction. Another b=0 image in anterior to posterior phase encoding direction was acquired for susceptibility distortion correction.

Image preprocessing

MRI data were all processed using the iDIO pipeline (https://github.com/iDIO4dMRI/iDIO)[5] that included functions of MRtrix3 (https://www.mrtrix.org/),[6] FSL (https://fsl.fmrib.ox.ac.uk/),[7] ANTs (http://stnava.github.io/ANTs/),[8] and PreQual

(https://github.com/MASILab/PreQual).[9] The DWIs preprocessing procedures included image denoising (MRtrix3 command: dwidenoise),[10, 11] Gibbs ringing removal (MRtrix3 command: mrdegibbs),[12] susceptibility-induced distortion, eddy current and subject movement correction (FSL command: topup and eddy), B1 field inhomogeneity correction (MRtrix3 command: dwibiascorrect)[13] and imaging resampling into 2 mm3 isotropic voxels. T1w image preprocessing includes Gibbs-ringing artifact removal (MRtrix3 command: mrdegibbs)[12] and B1 field inhomogeneity bias correction (ANTs command: N4BiasFieldCorrection command), both of which can improve skull stripping. A skull-stripped brain mask is generated using the antsBrainExtraction in ANTs. Next, image registration from T1 to DWI uses the boundary-based registration method (FLIRT in FSL).[14, 15] T1w brain mask is then transformed into DWI space for further usage. Last, using the brain mask with preprocessed DWIs, DWIs with b-values lower than 1500 s/mm2 are extracted and analyzed using the diffusion tensor model with weighted least square (FSL command: dtifit).[16]

Reference

1. Nasreddine ZS, Phillips NA, Bedirian V, et al. The Montreal Cognitive Assessment, MoCA: a brief screening tool for mild cognitive impairment. J Am Geriatr Soc 2005;53:695-699.

2. Tsai C-F, Lee W-J, Wang S-J, Shia B-C, Nasreddine Z, Fuh J-L. Psychometrics of the Montreal Cognitive Assessment (MoCA) and its subscales: validation of the Taiwanese version of the MoCA and an item response theory analysis. International Psychogeriatrics 2011;24:651-658.

3. Rosen WG, Mohs RC, Davis KL. A new rating scale for Alzheimer's disease. Am J Psychiatry 1984;141:1356-1364.

4. Wei YC, Chen CK, Lin C, et al. Normative Data of Mini-Mental State Examination, Montreal Cognitive Assessment, and Alzheimer’s Disease Assessment Scale-Cognitive Subscale of Community-Dwelling Older Adults in Taiwan. Dementia and Geriatric Cognitive Disorders 2022.

5. Hsu CH, Chong ST, Kung YC, Kuo KT, Huang CC, Lin CP. Integrated diffusion image operator (iDIO): A pipeline for automated configuration and processing of diffusion MRI data. Hum Brain Mapp 2023;44:2669-2683.

6. Tournier JD, Smith R, Raffelt D, et al. MRtrix3: A fast, flexible and open software framework for medical image processing and visualisation. Neuroimage 2019;202:116137.

7. Jenkinson M, Beckmann CF, Behrens TE, Woolrich MW, Smith SM. Fsl. Neuroimage 2012;62:782-790.

8. Avants BB, Tustison NJ, Song G. Advanced normalization tools (ANTS). Insight j 2009;2:1-35.

9. Cai LY, Yang Q, Hansen CB, et al. PreQual: An automated pipeline for integrated preprocessing and quality assurance of diffusion weighted MRI images. Magn Reson Med 2021;86:456-470.

10. Veraart J, Fieremans E, Novikov DS. Diffusion MRI noise mapping using random matrix theory. Magn Reson Med 2016;76:1582-1593.

11. Veraart J, Novikov DS, Christiaens D, Ades-Aron B, Sijbers J, Fieremans E. Denoising of diffusion MRI using random matrix theory. Neuroimage 2016;142:394-406.

12. Kellner E, Dhital B, Kiselev VG, Reisert M. Gibbs-ringing artifact removal based on local subvoxel-shifts. Magn Reson Med 2016;76:1574-1581.

13. Tustison NJ, Avants BB, Cook PA, et al. N4ITK: improved N3 bias correction. IEEE Trans Med Imaging 2010;29:1310-1320.

14. Jenkinson M, Smith S. A global optimisation method for robust affine registration of brain images. Med Image Anal 2001;5:143-156.

15. Jenkinson M, Bannister P, Brady M, Smith S. Improved optimization for the robust and accurate linear registration and motion correction of brain images. Neuroimage 2002;17:825-841.

16. Basser PJ, Mattiello J, LeBihan D. Estimation of the effective self-diffusion tensor from the NMR spin echo. J Magn Reson B 1994;103:247-254.
