# Supplementary material for: Vascular risk factors and astrocytic marker for the glymphatic system activity
Source: Radiol Med. 2023 Jul 18;128(9):1148–61. doi: 10.1007/s11547-023-01675-w (PMC10474179; doi:10.1007/s11547-023-01675-w)
Supplement: Supplementary file 2 — Supplementary file2 (PDF 140 KB) [file 11547_2023_1675_MOESM2_ESM.pdf]

eAppendix2

eTable 1. Correlation between DTI-ALPS and biomarkers, demographics, and laboratory data			
		**Pearson's *r* with DTI-ALPS**	***p***
Image marker			
DTI-ALPS index	1.24 ± 0.10	N/A	N/A
Plasma biomarkers			
GFAP (pg/mL)	17.06 ± 6.13	-0.201	0.093†
NfL (pg/mL)	9.35 ± 2.97	-0.066	0.587
Aβ42 (pg/mL)	16.35 ± 0.49	0.019	0.878
Aβ40 (pg/mL)	52.03 ± 6.51	0.068	0.574
Aβ42/Aβ40 ratio	0.32 ± 0.05	-0.087	0.470
Tau (pg/mL)	22.25 ± 2.81	0.022	0.854
p-Tau181 (pg/mL)	3.73 ± 0.59	-0.028	0.815
Demographics			
Age (year)	67.65 ± 3.75	-0.434	<0.001*,§
Education (year)	10.92 ± 3.68	0.019	0.874
Physiological measures			
SBP (mmHg)	130.42 ± 15.93	-0.106	0.386
DBP (mmHg)	75.99 ± 9.10	0.036	0.771
HR (beat per minute)	77.04 ± 10.11	0.058	0.640
BMI	24.80 ± 2.96	-0.295	0.014*
Weight	62.02 ± 10.27	-0.348	0.003*,§
Waist (cm)	83.06 ± 10.86	-0.455	<0.001*,§
Arm circumference (cm)	27.57 ± 3.07	-0.170	0.406
Calf circumference (cm)	34.13 ± 2.93	-0.280	0.028*
Thigh circumference (cm)	46.79 ± 4.83	-0.002	0.987
Lab data (blood)			
Cr	0.83 ± 0.23	-0.387	0.001*,§
BUN	16.47 ± 4.50	-0.303	0.011*
eGFR	80.27 ± 16.83	0.111	0.364
BUN/Cr ratio	20.38 ± 5.15	0.050	0.685
Na	142.23 ± 1.70	0.055	0.655
K	4.36 ± 0.44	-0.180	0.138
Ca	9.45 ± 0.30	-0.025	0.838
P	3.69 ± 0.50	0.300	0.012**
Estimated anion gap	123.59 ± 22.12	-0.322	0.007*
Albumin	4.63 ± 0.24	-0.035	0.776
Uric acid	5.48 ± 1.30	-0.381	0.001*,§
ALT	25.58 ± 17.21	0.033	0.789
AST	24.03 ± 10.41	0.091	0.457
ALK-P	75.00 ± 19.03	0.086	0.481
Bilirubin total	0.58 ± 0.23	-0.084	0.496

Glucose AC	100.10 ± 16.51	-0.264	0.028[*]
HbA1c	5.93 ± 0.77	-0.324	0.007[*]
HOMA-IR	2.31 ± 1.19	0.142	0.245
Triglyceride	120.49 ± 54.93	-0.342	0.004[*,§]
Tchol	192.68 ± 34.83	0.292	0.015[**]
HDL	59.61 ± 17.51	0.396	0.001[**,§]
LDL	117.84 ± 30.27	0.193	0.112
VLDL	24.12 ± 11.02	-0.342	0.004[*,§]
Tchol/HDL ratio	3.44 ± 0.94	-0.277	0.021[*]
WBC	5.54 ± 1.46	-0.010	0.938
Neutrophil/lymphocyte ratio	1.93 ± 1.17	0.050	0.685
RBC	4.67 ± 0.62	-0.207	0.087
Hemoglobin	13.62 ± 1.51	-0.236	0.051
Hematocrit	40.81 ± 4.21	-0.219	0.071
Platelet	243.68 ± 63.60	0.207	0.087
hs-CRP	1.72 ± 1.96	-0.163	0.201
Ferritin	245.74 ± 198.07	-0.238	0.150
Folate	10.85 ± 5.29	0.033	0.787
Vitamin B12	792.45 ± 451.78	-0.029	0.815
Vitamin D	25.64 ± 7.54	-0.212	0.080
Laboratory test (urine)			
Microalbumin	4.36 ± 10.70	-0.038	0.772
Urine albumin/Cr ratio	30.19 ± 69.91	-0.104	0.426
Cognitive test			
MoCA	25.83 ± 2.89	0.140	0.244
ADAS-cog	6.10 ± 3.37	-0.139	0.248
DSST	56.49 ± 15.74	0.248	0.037[**]
DST-f	12.06 ± 2.31	0.041	0.733
DST-b	6.80 ± 2.57	-0.048	0.692
FMT	36.37 ± 4.24	0.272	0.022[**]
LNS	9.18 ± 2.30	0.044	0.716
CF-animal	18.42 ± 5.06	-0.211	0.077
CF-fruit	13.56 ± 2.94	0.188	0.117
CF-city	21.24 ± 5.62	-0.148	0.217
CF-color	13.52 ± 3.83	0.083	0.490
Mental assessment			
HADS-A	5.54 ± 4.41	0.238	0.046[**]
HADS-D	4.89 ± 3.86	0.158	0.189
Physical activity			
IPAQ-SF MET (per week)	4172 ± 2477	0.104	0.390
Sleep questionnaire			
PSQI total	8.65 ± 4.45	0.195	0.104
ESS	4.69 ± 4.14	0.278	0.019[**]
SOMNOwatch sleep profile[¶]			
Total sleep time (minute)	215.5 ± 86.5	0.139	0.425

Total sleep period (minute)	381.9 ± 77.8	0.051	0.769
Sleep efficiency (%)	50.82 ± 19.31	0.116	0.506
Sleep latency (minute)	24.6 ± 38.7	-0.176	0.312
REM sleep (minute)	75.6 ± 53.0	-0.003	0.985
NREM sleep (minute)	139.9 ± 75.1	0.163	0.350
N1 (minute)	8.1 ± 8.9	-0.136	0.436
N2 (minute)	102.1 ± 60.9	0.128	0.462
N3 (minute)	12.6 ± 8.7	0.206	0.235
N4 (minute)	16.3 ± 19.8	0.181	0.298
NREM/REM ratio	7.13 ± 14.13	0.301	0.078

Data are presented as mean ± SD. [*] A negative correlation with statistical significance at a 2-tailed *p*-value < 0.05. [**] A significant positive correlation with a $p < 0.05$ (2-tailed). [¶] 35 participants completed the 72-hr actigraphy and sleep staging by SOMNOWatch. [†] One-tailed t-test with a $p = 0.046$ under the assumption that astrocytic injury with increased release of GFAP into blood was related to a decrease in glymphatic function. [§] FDR-corrected $p < 0.05$. After applying the false discovery rate (FDR) to correct for multiple comparisons, the following variables passed FDR correction: age, body weight, waist, creatinine, uric acid, triglyceride, HDL, and VLDL.

Abbreviations: DTI-ALPS, Diffusion tensor image analysis along the perivascular space; GFAP, glial fibrillary acidic protein; NfL, neurofilament light chain; Aβ42, beta-amyloid 42; Aβ40, beta-amyloid 40; Tau, total tau; p-Tau181, phosphorylated tau 181; SBP, systolic blood pressure; DBP, diastolic blood pressure; HR, heart rate; BMI, body mass index; Cr, creatinine; BUN, blood urea nitrogen; eGFR, estimated Glomerular filtration rate; ALT, alanine transaminase; AST, aspartate transaminase; ALK-P, alkaline phosphatase; HbA1c, glycated hemoglobin; HOMA-IR, homeostatic model assessment - insulin resistance; Tchol, total cholesterol; HDL, high-density lipoprotein; LDL, low-density lipoprotein; VLDL, very-low-density lipoprotein; WBC, white blood cell; RBC, red blood cell; hs-CRP, high-sensitivity C-reactive protein; MoCA, Montreal Cognitive Assessment; ADAS-cog, Alzheimer’s Disease Assessment Scale; DSST, Digit Symbol Substitution Test; DST-f, Digit Span Test forward; DST-b, Digit Span Test backward; FMT, Facial Memory Test; LNS, Letter Number Sequencing; CF, Category Fluency; HADS-A, Hospital Anxiety and Depression Scale - anxiety subscale; HADS-D, Hospital Anxiety and Depression Scale - depression subscale; IPAQ-SF MET, International Physical Activity Questionnaire-Short From metabolic equivalent minutes per week; PSQI, Pittsburg Sleep Quality Index; ESS, Epworth Sleepiness Scale; REM, rapid eye movement; NREM, non-rapid eye movement; FDR, false discovery rate.

eTable 2. DTI-ALPS group differences of demographics and multidomain classes				
	**N**	**DTI-ALPS**	**SMD (95% CI)**	***p***
Demographics				
Sex (female/male)	44/27	1.283±0.085/1.183±0.105	1.0769 (0.566, 1.5877)	<0.001*,§
Lifestyle				
Smoking habit (Y/N)	20/50	1.199±0.117/1.262±0.095	-0.6292 (-1.1581, -0.1002)	0.020*
Alcohol consumption (Y/N)¶	8/59	1.254±0.06/1.241±0.107	0.1221 (-0.6167, 0.8608)	0.641
Tea user (Y/N)	38/32	1.220±0.108/1.273±0.095	-0.5146 (-0.9926, -0.0367)	0.036*
Coffee user (Y/N)	47/23	1.245±0.104/1.243±0.109	0.0262 (-0.4726, 0.525)	0.919
Chronic illness				
Hypertension (Y/N)	31/39	1.230±0.104/1.256±0.106	-0.2510 (-0.7244, 0.2225)	0.301
Diabetes mellitus (Y/N)	12/58	1.180±0.100/1.258±0.102	-0.7662 (-1.4006, -0.1318)	0.018*
Chronic kidney disease (Y/N)	15/53	1.226±0.128/1.250±0.099	-0.2199 (-0.7943, 0.3545)	0.455
Physiological measures				
Overweight (BMI≥25) (Y/N)	34/35	1.213±0.108/1.272±0.096	-0.5818 (-1.0636, -0.0999)	0.018*
Metabolic syndrome (Y/N)†	17/53	1.197±0.112/1.259±0.099	-0.6068 (-1.1623, -0.0513)	0.033*
Sleep questionnaire				
Poor sleep quality (PSQI≥5/<5)	57/14	1.256±0.096/1.198±0.127	0.5733 (-0.0189, 1.1655)	0.124

* Statistical significance at a 2-tailed *p*-value < 0.05. ¶ The alcohol consumption considered harmful alcohol use with an Alcohol Use Disorders Identification Test (AUDIT) score ≥ 5 points (https://auditscreen.org). † Having metabolic syndrome was defined by ≥ 3 positive items over the five criteria of metabolic syndrome [1]. § FDR-corrected $p < 0.05$. Out of the binary variables, sex passed the false discovery rate (FDR) correction for multiple comparisons. Abbreviations: BMI, body mass index; PSQI, Pittsburgh Sleep Quality Index; SMD, standardized mean difference; 95% CI, 95% confidence interval.

Reference

1. Alberti KG, Eckel RH, Grundy SM, Zimmet PZ, Cleeman JI, Donato KA, Fruchart JC, James WP, Loria CM, Smith SC, Jr., et al: **Harmonizing the metabolic syndrome: a joint interim statement of the International Diabetes Federation Task Force on Epidemiology and Prevention; National Heart, Lung, and Blood Institute; American Heart Association; World Heart Federation; International Atherosclerosis Society; and International Association for the Study of Obesity.** *Circulation* 2009, **120:**1640-1645.
